# Psychometric Properties of the Copenhagen Burnout Inventory in a Sample of Medical Students in Kazakhstan

**DOI:** 10.11621/pir.2021.0202

**Published:** 2021-06-30

**Authors:** Aidos K. Bolatov, Telman Z. Seisembekov, Altynay Zh. Askarova, Bakhyt Igenbayeva, Dariga S. Smailova, Hengameh Hosseini

**Affiliations:** a Astana Medical University, Nur-Sultan, Kazakhstan; b Kazakhstan’s School of Public Health, Almaty, Kazakhstan; c University of Scranton, Scranton, PA, USA

**Keywords:** burnout, medical students, Kazakhstan, Copenhagen Burnout Inventory, validation

## Abstract

**Background:**

The Copenhagen Burnout Inventory (CBI) has demonstrated good psychometric properties among different populations, but there is no known data on its validity among Russian-speaking medical students. The CBI-Student Survey focuses only on fatigue, but measures exhaustion in four different life domains: Personal Burnout (PB), Studies-Related Burnout (SRB), Colleague-Related Burnout (CRB), and Teacher-Related Burnout (TRB).

**Objective:**

To investigate the psychometric properties of the Russian version of the Copenhagen Burnout Inventory–Student Survey (R-CBI-S).

**Design:**

A cross-sectional study was carried out among 771 medical students at Astana Medical University (Nur-Sultan, Kazakhstan). Statistical analyses included test-retest reliability, internal consistency, item analysis, convergent and concurrent validity, and confirmatory factor analysis. Concurrent validity was evaluated by bivariate correlations of R-CBI-S with anxiety, depression, and satisfaction with the study.

**Results:**

Test-retest reliability showed an ICC of 0.81. All item-total correlations for the total scale were positive (range 0.31–0.76). The Cronbach’s alpha coefficient was 0.94 (0.896 for PB, 0.884 for SRB, 0.874 for CRB, and 0.926 for TRB). The Barlett’s sphericity test result was significant (p < 0.001), and the KMO measure of sampling adequacy exceeded 0.947. Convergent validity analysis results: PB (AVE = 0.52, CR = 0.87), SRB (AVE = 0.50, CR = 0.87), CRB (AVE = 0.51, CR = 0.86), TRB (AVE = 0.56, CR = 0.88). The R-CBI-S achieved good levels of goodness-of-fit indices (RMSEA = 0.0611; CFI= 0.940; TLI = 0.933).

**Conclusion:**

The test results indicated that the R-CBI-S scale appears to be a reliable and valid instrument. The R-CBI-S may be a useful tool in future research to identify burnout factors based on specific life domains for developing effective prevention measures among medical students.

## Introduction

Burnout is a growing epidemic among medical students, which has been shown to have psychological and performance-related detriments (Bullock et al., 2017). Medical students are not only more likely to be burned out compared to the general population, but are increasingly likely to suffer burnout as they advance in their medical training (Dyrbye et al., 2014; Dyrbye et al., 2006). This often leads to significant psychological changes that manifest as depression, insomnia, substance abuse disorders, poor physical health, psychosomatic conditions, relational problems, social withdrawal, and professional dysfunction (Aguiar et al., 2009; Almeida et al., 2016). Burnout can also affect medical students’ will to continue to espouse professional qualities, such as honesty, integrity, altruism, and self-regulation (Dyrbye et al., 2010). Based on these findings, it is clear that burnout is a serious problem in the training and professional development of medical students.

Several methods have been developed to study burnout among students, namely the Maslach Burnout Inventory–General Survey for Students (MBI-SS; Maslach, Jackson, & Leiter, 2017), the Oldenburg Burnout Inventory for college students (OLBI-S; Demerouti & Bakker, 2008), and the Copenhagen Burnout Inventory (CBI) proposed by Kristensen, Borritz, Villadsen, and Christensen (2005). The MBI-SS assesses the prevalence of burnout based on subjects’ emotional exhaustion, depersonalization, and reduced professional satisfaction and effectiveness, as captured by 22 items. The OLBI-S includes two dimensions, exhaustion and disengagement, with the distinction that it captures exhaustion across physical, affective, and cognitive dimensions compared to the single emotional dimension measured by the MBI-SS (Demerouti & Bakker, 2008). By comparison, CBI focuses only on fatigue/emotional exhaustion, but measures the respondent’s attribution of this exhaustion to three different life domains: Personal Burnout, Work-Related Burnout, and Client-Related Burnout (Molinero Ruiz, Basart Gómez-Quintero, & Moncada Lluis, 2013). The CBI measures burnout in a more straightforward way (Yeh, Cheng, Chen, Hu, & Kristensen, 2007). According to one systematic review of the CBI and the OLBI, the quality of evidence for sufficient content validity was moderate, while for the MBI it was very low. Moreover, the CBI was more appropriate for valid and reliable use in medical research and practice (Shoman et al., 2021). A systematic review and meta-analysis conducted among midwives showed that the CBI addressed more realistically the levels of physical and mental exhaustion and was very useful (Suleiman-Martos et al., 2020).

In recent years, the CBI has been validated in different countries and study populations, such as university professors and academic staff members at Brazilian public universities (Rocha et al., 2020), an academic healthcare institution sample in the U.S. (Thrush, Gathright, Atkinson, Messias, & Guise, 2020), Greek doctors (Papaefstathiou, Tsounis, Malliarou, & Sarafts, 2019), Iranian nurses (Mahmoudi et al., 2017), Korean homecare workers (Jeon, You, Kim, Kim, & Cho, 2019), and U.S. nurses (Montgomery, Azuero, & Patrician, 2021). In all cases, the CBI demonstrated adequate validity and reliability for measuring burnout. Andrew Chin et al. (2018) investigated the validity of the CBI among Malaysian medical students, but using the original three-dimensional structure. Campos, Carlotto, and Marôco (2013) adapted the CBI original inventory for students as the CBI–Student Survey, and developed items measuring students’ Personal Burnout, Studies-Related Burnout, Colleague-Related Burnout, and Teacher-Related Burnout.

This study aims at evaluating the reliability and validity of the Russian version of the Copenhagen Burnout Inventory–Student Survey (R-CBI-S) in a sample of medical students at the Astana Medical University, Kazakhstan.

## Methods

### Participants

All medical students at any stage of their medical education at Astana Medical University were eligible to participate. Participants were invited via the “messenger” app and the university’s information portal, Sirius, to fill out an online questionnaire created on the 1ka platform (www.1ka.si) during the period October–December 2019. The questionnaire was completed by 771 students (response rate 40%). Of the participants, 25.0% were male. Academic year distribution among students was 1 year (218), 2 year (137), 3 year (125), 4 year (62), 5 year (60), and 6 year (169). The average age of the respondent was 20.7 years (ranged in age from 18 to 33).

### Procedure

The Copenhagen Burnout Inventory–Student Survey was converted into the Russian language from the original English version using a forward-backward translation process performed by specialists in the field of psychology and language. The final questionnaire was revised based on feedback from a sample of 20 participants through a pilot study.

Data analysis was conducted using Microsoft Excel 2007, SPSS version 20.0, and Jamovi version 1.2.17. A statistically significant difference was accepted at a p-value of less than 5%.

The reliability of the scale (performed on a sample of 20 subjects during a two-week interval) was evaluated using the intraclass correlation coefficient (ICC). According to Koo and Li (2016) ICC values between 0.75 and 0.9 indicate good reliability, and values greater than 0.90 indicate excellent reliability.

Internal consistency was evaluated by the total scale and subscales reliability analysis reflected by Cronbach’s alpha coefficient. A Cronbach’s alpha coefficient with a value of ≥ 0.7 is acceptable (Taber, 2018). Corrected item-total correlation was carried out.

Convergent validity was checked with average variance extracted (AVE) and composite reliability (CR). Values of 0.5 or more for AVE and 0.6 or more for CR were considered as having significant convergent validity (Kline, 2011). Concurrent validity was evaluated by bivariate correlations of R-CBI-S with anxiety (GAD-7; Spitzer, Kroenke, Williams, & Lowe, 2006), depression (PHQ-9; Kroenke & Spitzer, 2002), and satisfaction with the study.

Construct validity was established by the confirmatory factor analysis (CFA) technique, with Bartlett’s test of sphericity and the Kaiser-Meyer-Olkin (KMO) measure of sampling adequacy used to test the dataset for factor analysis suitability. The CFA is used to assess the overall goodness of fit: the Root Mean Square of Error Approximation RMSEA (< 0.08); the Comparative Fit Index CFI(> 0.9); and the Tucker-Lewis Index TLI (> 0.9) (Xia & Yang, 2019).

### Questionnaire

The Russian version of the Copenhagen Burnout Inventory was adapted for students. The R-CBI-S consists of 25 items that represent four dimensions: Personal Burnout (PB) — 6 items (numbers 1, 2, 3, 4, 5, and 6), Studies-Related Burnout (SRB) — 7 items (numbers 7, 8, 9, 10, 11, 12, and 13), Colleague-Related Burnout (CRB) — 6 items (numbers 14, 15, 16, 17, 18, and 19), and Teacher-Related Burnout (TRB) — 6 items (numbers 20, 21, 22, 23, 24, and 25). The answers that can be given to each item are “always,” “frequently,” “sometimes,” “rarely,” and “never.” The scores attributed to these answers are 100, 75, 50, 25, and 0% respectively, with inverse scoring for item 10. For each scale, a total average score was calculated. According to Kristensen’s criteria of burnout levels, scores of 50 to 74 are considered moderate, 75–99 high, and a score of 100 is considered severe burnout (Borritz et al., 2006).

## Results

The final translated Russian version of the R-CBI-S is presented in *Table 1*.

The test-retest reliability showed an ICC of 0.81 (CI 95% 0.63–0.94) for the R-CBI-S. The overall Cronbach’s alpha coefficient of the R-CBI-S was 0.939 (0.896 for PB, 0.884 for SRB, 0.874 for CRB, and 0.926 for TRB), which indicates a high level of internal consistency. Corrected item-total correlation is shown in Table 1. All item-total correlations for the total scale were positive (range 0.31–0.76) within the criterion of the item-total correlation greater than 0.30 (DeVellis, 2003).

**Table 1:** Russian version of the Copenhagen Burnout Inventory–Student Survey. Cronbach’s alpha internal consistency and convergent validity analysis

Item	R-CBI-S	Corrected item-total correlation	α if item deleted
**Personal Burnout**			
Cronbach’s alpha = 0.896, AVE = 0.52, CR = 0.87			
1	How often do you feel tired? *Как часто Вы чувствуете усталость?*	0.599	0.937
2	How often are you physically exhausted? *Как часто Вы физически истощены?*	0.603	0.937
3	How often are you emotionally exhausted? *Как часто Вы эмоционально истощены?*	0.680	0.936
4	How often do you think: “I can’t take it anymore”? *Как часто Вы думаете: «Я не могу больше этого терпеть»?*	0.700	0.935
5	How often do you feel worn out? *Как часто Вы чувствуете себя измотанным?*	0.672	0.936
6	How often do you feel weak and susceptible to illness? *Как часто Вы чувствуете себя слабым и восприимчивым к болезни?*	0.584	0.937
**Studies-Related Burnout**			
Cronbach’s alpha = 0.884, AVE = 0.50, CR = 0.87			
7	Do you feel worn out at the end of the working day? *Чувствуете ли Вы усталость в конце учебного дня?*	0.595	0.937
8	Are you exhausted in the morning at the thought of another day at work? *Вы чувствуете истощенность по утрам от мыслей о новом дне на учебе?*	0.688	0.935
9	Do you feel that every working hour is tiring for you? *Вы чувствуете, что каждый учебный час утомляет Вас?*	0.703	0.935
10	*Do you have enough energy for family and friends during leisure time? *У Вас достаточно энергии для семьи и друзей в свободное время?	0.435	0.939
11	Are your studies emotionally exhausting? *Является ли Ваша учеба эмоционально истощающей?*	0.725	0.935
12	Do your studies frustrate you? *Расстраивает ли Вас ваша учеба?*	0.643	0.936
13	Do you feel burnout because of your studies? *Вы чувствуете себя выгоревшим из-за учебы*	0.757	0.934
**Colleague-Related Burnout**			
Cronbach’s alpha = 0.874, AVE = 0.51, CR = 0.86			
14	Do you find it hard to work with colleagues? *Вам тяжело работать с коллегами?*	0.525	0.938
15	Does it drain your energy to work with colleagues? *Вы тратите силы на работу с коллегами?*	0.308	0.940
16	Do you find it frustrating to work with colleagues? *Вам неприятно работать с коллегами?*	0.451	0.939
17	Do you feel that you give more than you get back when you work with colleagues? *Вы чувствуете, что отдаете больше, чем получаете, когда работаете с коллегами?*	0.330	0.940
18	Are you tired of working with colleagues? *Вы устали работать с коллегами?*	0.496	0.938
19	Do you sometimes wonder how long you will be able to continue working with colleagues? *Вы иногда задаетесь вопросом, как долго вы сможете продолжать работать с коллегами?*	0.469	0.938
**Teacher-Related Burnout**			
Cronbach’s alpha = 0.926, AVE = 0.56, CR = 0.88			
20	Do you find it hard to work with teachers? *Вам тяжело работать с преподавателем?*	0.702	0.935
21	Does it drain your energy to work with teachers? *Вы тратите силы на работу с преподавателем?*	0.589	0.937
22	Do you find it frustrating to work with teachers? *Вам неприятно работать с преподавателем?*	0.676	0.936
23	Do you feel that you give more than you get back when you work with teachers? *Вы чувствуете, что отдаете больше, чем получаете, когда работаете с преподавателем?*	0.607	0.937
24	Are you tired of working with teachers? *Вы устали работать с преподавателем?*	0.728	0.935
25	Do you sometimes wonder how long you will be able to continue working with teachers? *Вы иногда задаетесь вопросом, как долго вы сможете продолжать работать с преподавателем?*	0.709	0.935

Note. * Reversed item.

**Figure 1.**
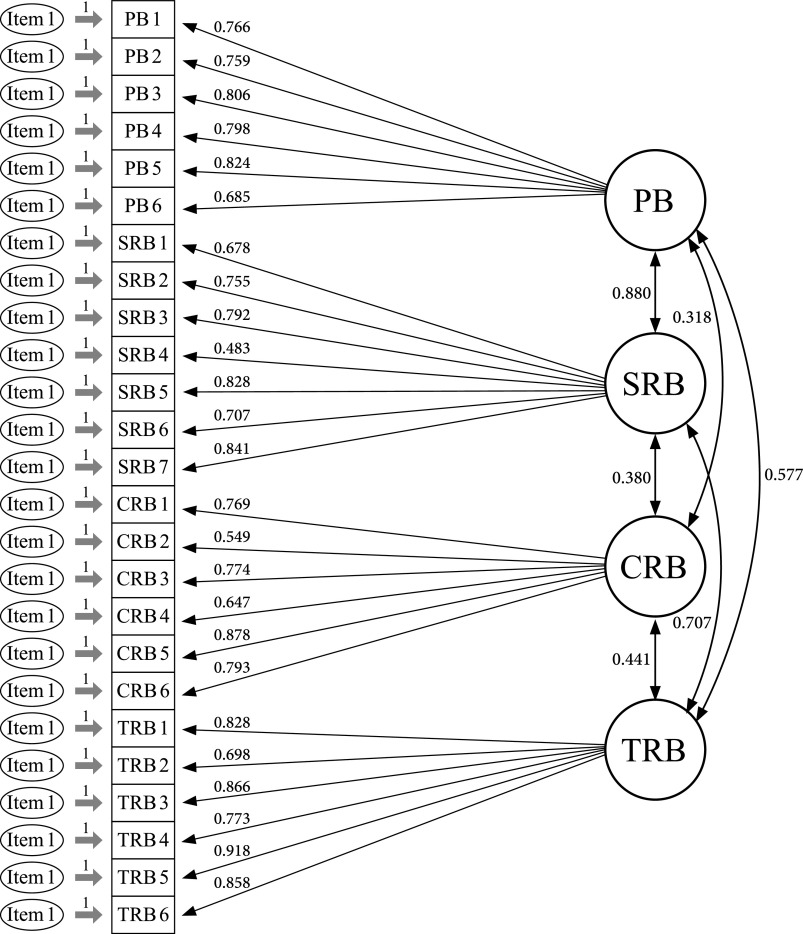
Confirmatory Factor Analysis of the Russian version of the Copenhagen Burnout Inventory–Student Survey (R-CBI-S) [χ2/df = 3.881; CFI= 0.940; TLI = 0.933; RMSEA = 0.0611]

The Barlett’s sphericity test result was significant (p < 0.001), and the KMO measure of sampling adequacy exceeded 0.947. Extracted AVE and CR from convergent validity analysis showed in Table 1. According to the CFA analysis, the model fit of the four-factor R-CBI-S model was confirmed by the indices: χ2/df 3.881; RM-SEA = 0.0611; CFI= 0.940; TLI = 0.933, with cumulative variance at 59.5% (by comparison, a one-factor model showed χ2/df = 17.963; RMSEA = 0.148; CFI= 0.638; TLI = 0.605). *Figure 1* shows the factor model. Analysis of the eigenvalues indicated that four factors extracted with values above 1.0 (9.76 for TRB, 2.33 for the CRB, 1.44 for the SRB, and 1.12 for the PB) according to Henson & Roberts (2006).

*Table 2* shows the correlations of the R-CBI-S with other variables. Weak positive correlations were found for R-CBI-S and GAD-7, PHQ-9. Satisfaction with the study was found to be negatively associated with R-CBI-S.

**Table 2:** Concurrent validity of the R-CBI-S

Variables	(1)	(2)	(3)	(4)
Satisfaction with study (1)	–			
GAD-7 (2)	–0.203	–		
PHQ-9 (3)	–0.342	0.560	–	
R-CBI-S (4)	–0.237	0.412	0.419	–

*Note. All correlations are significant at p < 0.001*.

The total R-CBI-S mean score was 39.96, and the mean subscale scores for this sample were 52.62 (PB), 50.93 (SRB), 23.50 (CRB), and 32.77 (TRB).

## Discussion

Analysis of the literature showed that burnout is an important component of medical students’ mental health, which can affect the learning process and have further professional consequences. A feature of the CBI is that it divides burnout into four components. This makes it possible to identify predictors of burnout covering not only exhaustion, but dividing it into personal, studies-related, colleague-related, and teacher-related burnout, ultimately to draw up a more comprehensive approach to organization of the educational process. We agree with the opinion of Sedlar, Šprah, Tement, and Socan (2015), that before using the scale, one must go through a validation process to obtain the most reliable results.

The purpose of this study was to examine the psychometric properties of the Russian version of the CBI-S. Following adaptation and psychometric tests, this study found that the survey was reliable and valid for assessing burnout among Russian-speaking medical students in Kazakhstan. The ICC analysis showed that the R-CBI-S had high stability within 2 weeks of the test-retest (mean ICC 0.81).

The internal consistencies of the four subscales were satisfactory, with all the Cronbach’s alpha values ranging from 0.874 to 0.926, and Cronbach’s alpha for R-CBI-S being 0.939. These results are slightly lower than those reported by Campos et al. (2013), with Cronbach’s alpha ranging from 0.875 to 0.931, and 0.957 for the CBI-S. The results of the current study present good internal consistency values. The corrected item-total correlation values obtained for the items are relatively high, which demonstrates that the items of R-CBI-S are relatively homogeneous and are measuring the same overall construct.

AVE for all dimensions was equal to or more than 0.5, suggesting an adequate level of convergent validity. The CR values of the R-CBI-S constructs ranged between 0.86 and 0.88, which indicates a high level of convergent validity.

The R-CBI-S was associated with anxiety, depression, and satisfaction with the study, lending support to the scale’s concurrent validity. A validated Chinese version of CBI was correlated not only with anxiety and depression, but also with physical distress and social support (Fong, Ho, & Ng, 2013).

The R-CBI-S demonstrated satisfactory construct validity, as tested by CFA. The results indicated that most fit indices were in acceptable ranges. Sufficiency of the model was demonstrated by Bartlett’s test of sphericity and the KMO measure.

## Conclusion

The R-CBI-S appears to be a reliable and valid instrument in measuring medical students’ burnout. The instrument could be useful for future efforts to develop an effective preventive intervention for burnout syndrome determination among Russian-speaking medical students.
